# Getting to the Bottom of Saddle Sores: A Scoping Review of the Definition, Prevalence, Management and Prevention of Saddle Sores in Cycling

**DOI:** 10.3390/ijerph19138073

**Published:** 2022-06-30

**Authors:** Daniel Napier, Neil Heron

**Affiliations:** 1 School of Medicine, Dentistry and Biomedical Sciences, Queen’s University Belfast, 97 Lisburn Road, Belfast BT9 7BL, UK; 2 Centre for Public Health, Queen’s University Belfast, Belfast BT9 7BL, UK; n.heron@qub.ac.uk

**Keywords:** saddle sores, PNI, perineal nodular induration, cycling

## Abstract

**Objectives:** To summarise and map the existing evidence relating to the definition, prevalence, prevention and management of saddle sores within the literature and highlight research gaps. **Design:** Scoping review. **Data Sources:** Three databases were searched using an appropriate search strategy agreed on by the authors with the aid of an experienced medical librarian; these databases were MEDLINE, EMBASE and Web of Science. **Eligibility Criteria:** To be included in this review, studies must have made specific reference to dermatological conditions that affect the saddle area, specifically arising from cycling, in either sex. **Results**: Seventeen studies were selected for inclusion. Saddle sores in males were the focus of thirteen studies, with only two reporting in females. Saddle sores were defined as connective tissue lesions affecting the skin in the saddle area, which can be both acute and chronic. Commonly cited preventions were chamois cream, high quality, well-fitting cycling equipment and good personal hygiene. Management in the early stages usually involves rest. Topical and intralesional steroids and lubricating creams are recommended treatments for small saddle sores, with surgical excision an option for larger, persistent saddle sores. However, surgery and steroid use may increase risk of recurrence. **Conclusions:** Saddle sores are an underrepresented, male-dominated issue within the literature. There is particularly limited evidence around treatment options, including topical steroids and surgical removal. Further well-designed observational studies and/or randomised controlled trials will help provide further evidence on prevalence, prevention and treatment available in the future.

## 1. Introduction

Saddle sores are skin lesions that occur in the area of the body in contact with the bicycle saddle during cycling [1]. This region includes the inner thighs, perineum, genitals and buttocks, and is known as the ‘saddle area’ [1]. Saddle sores are one of a wide range of conditions linked to prolonged contact with the bicycle saddle [1,2]. They are a reportedly common occurrence at both elite and recreational levels [1]. People who cycle frequently or over long-distances appear to be more at risk [3,4,5].

Pain and associated restriction of movement caused by saddle sores can negatively influence a cyclist’s participation in the sport [1]. Cycling is not only popular as a sport, but also as a means of transport. Saddle sores can affect commuter cyclists as well as athletes [3]. Recent evidence shows that cycling’s popularity is increasing, especially at a population level [6], putting saddle sores into context as a public health issue. Physical activity is important for both physical and mental health and can serve as a protective factor against cardiovascular disease, diabetes mellitus, obesity and depression, as well as numerous other health conditions [7,8]. Saddle sores can reduce physical activity levels in affected individuals [1].

Microtrauma to the skin caused by pressure and friction during cycling provides the stimulus for saddle sores to develop, as well as associated chafing [9]. Saddle sores are often associated with infections such as folliculitis and furunculitis, as heat and moisture from sweat create conditions for bacterial and fungal growth [1]. If saddle sores fail to resolve, they can develop into a chronic form, most commonly known as perinodular indurations (PNIs) [10].

Despite the apparent prevalence of this condition from the testimony of cyclists and the presence of articles on online websites and forums [1], there is very little scientific evidence on the exact definition, prevalence, management or prevention in the literature [1]. A recent scoping review regarding research into saddle sores in female competitive/elite athletes [1] was carried out, but, with the popularity of cycling in both sexes and in the general population, there is a need for a scoping review that summarises the current available evidence in terms of prevalence, prevention and treatment for cyclists of both genders at the recreational and elite levels [1]. In particular, there is a need for a common definition of the term “saddle sore” as significant variation can be observed within both the literature and online forums. This will help medical professionals to accurately diagnose and treat this condition, while allowing a coherent prevention message to be delivered to the cycling community.

The cultural sensitivity of this area of the body means that saddle sores can cause embarrassment, creating difficulties for athletes to have open dialogue about this issue with their healthcare professionals and support staff. A lack of knowledge from health professionals in identifying saddle sores and how to manage them means that cyclists may be reluctant to discuss this with practitioners, e.g., general/family practitioners (GPs). We therefore plan to undertake a scoping review to summarise and map the existing evidence relating to the definition, prevalence, prevention and management of saddle sores within the literature and highlight potential research gaps.

## 2. Methods

This scoping review was conducted in accordance with the Preferred Reporting Items for Systematic Reviews and Meta-analysis extension for scoping reviews (PRISMA-ScR) reporting guidelines. The five-stage process scoping review methodological framework by Arksey and O’Malley [11] was also utilized.

### 2.1. Stage 1: Identifying the Research Question

The research team included (D.N.) and (N.H.). After discussion, the team decided that a broad research question was needed to highlight the gaps in the research regarding saddle sores. The justification was given that there was a lack of robust evidence available on saddle sores in the literature, given the results of a preliminary search and the recommendations of a recent systematic scoping review [1]. Our research question was: What is the state of current evidence on the prevalence, prevention and management of saddle sores in cycling?

### 2.2. Stage 2: Identifying Relevant Studies

Inclusion criteria were:

- Articles written in English;
- Peer-reviewed published articles (widened if insufficient papers);
- Articles that include specific reference to skin conditions affecting the saddle area;
- Articles related to dermatological saddle conditions in both genders;
- Articles related to dermatological saddle conditions arising in either elite or recreational settings;
- Articles related to dermatological saddle conditions, specifically arising from cycling.

Exclusion criteria were:

- Articles not published;
- Inability to access full-text article;
- Grey literature.

Search strategy and databases: A literature search was conducted on 18 November 2021 to identify relevant studies. No date limit was used. The following databases were searched: MEDLINE, EMBASE and Web of Science. With input from an experienced medical librarian (R.F.), detailed search strategies for each database were decided upon. The final search strategy is detailed below. Further hand searching was performed for the included studies by reviewing their reference lists, as well as an initial Google Scholar search.

**Search Strategy**
**MEDLINE**, **EMBASE**

1. saddle sore*.mp.
2. perineum/
3. perineal health.mp.
4. perineal.mp.
5. saddle*.mp.
6. folliculitis/
7. bicycling/
8. cycli*.mp.
9. cyclist’s nodule.mp.
10. third testicle.mp.
11. perineal nodule.mp.
12. nodular induration.mp.
13. perineal abscess.mp.
14. biker’s nodule.mp.
15. perineal nodular induration.mp.
16. nodular swelling.mp.
17. perineal mass.mp.
18. abscess*.mp.
19. nodule*.mp.
20. 1 or 2 or 3 or 4 or 5 or 6 or 9 or 10 or 11 or 12 or 13 or 14 or 15 or 16 or 17 or 18 or 19
21. 7 or 8
22. 20 and 21
23. limit 22 to (english language and humans)

**Web of Science**

TOPIC: (saddle* OR perineum OR “perineal health” OR folliculitis OR perineal nodular induration OR perineal abscess OR biker’s nodule OR nodular swelling OR perineal mass OR cyclist’s nodule OR third testicle OR abscess OR nodule ) AND

TOPIC: (bicycl* or cycl* or bike)

### 2.3. Stage 3: Study Selection

The titles and abstracts of studies obtained through the search were independently reviewed against the eligibility criteria by the two authors. Based on the titles and abstracts, studies were sorted into ‘include’, ‘exclude’ or ‘uncertain’ groups. Articles which did not meet the eligibility criteria were discarded and the reasons for their exclusion were clearly documented. Any disagreements on study selection were resolved through discussion between the two reviewers, with a third reviewer available if required. The full texts of all the included and ‘uncertain’ publications were retrieved and these publications had their full contents screened against the eligibility criteria.

### 2.4. Stage 4: Charting the Data

Elements of the data were extracted by the primary reviewer (DN), and the accuracy of this data extraction was reviewed by the second data reviewer (NH). Meetings between the two reviewers resolved any issues with data extraction. Data extraction categories included:

- Author;
- Year of publication;
- Journal/Source of publication;
- Region of publication;
- Aims/Purpose of the study;
- Study population;
- Study design;
- Intervention type and duration (if applicable);
- Outcome measures and details of these (if applicable); and,
- Important results that relate to the scoping review research question above.

### 2.5. Stage 5: Collating, Summarising and Reporting the Results

**Figure 1:**
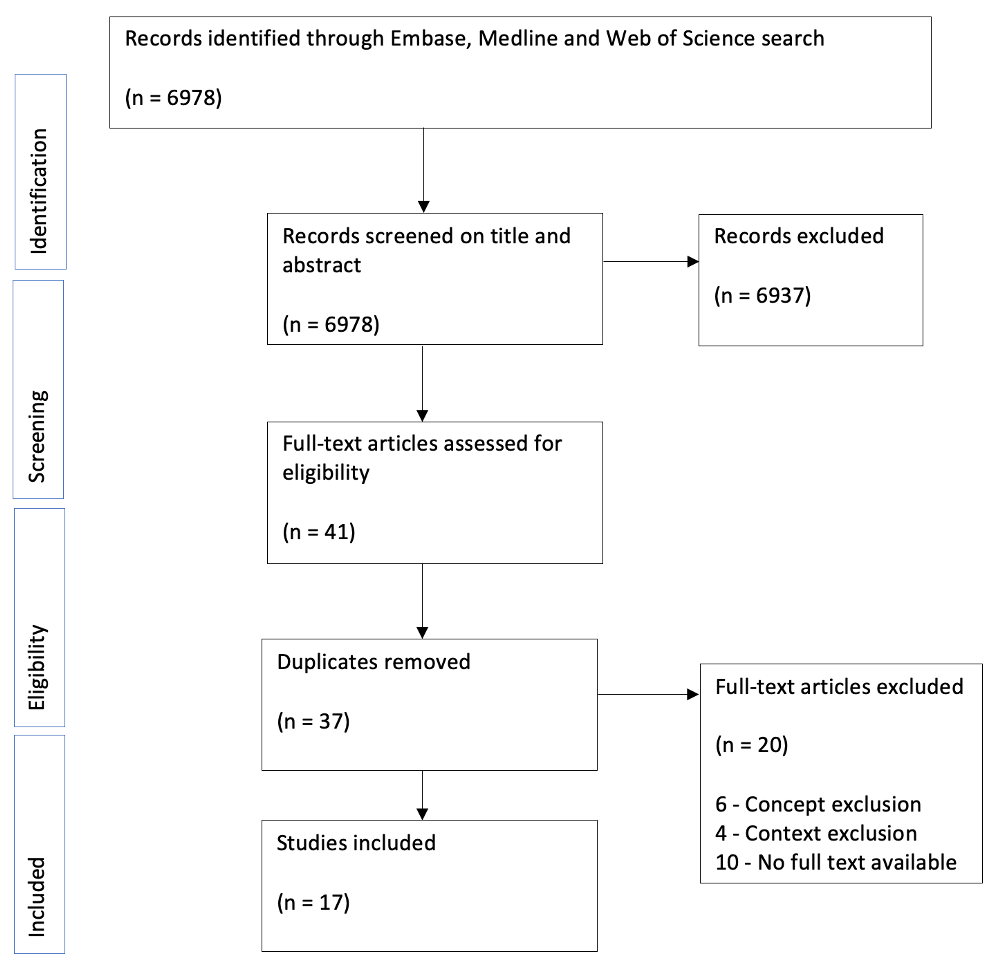
PRISMA flow diagram of study selection [12].

**Figure 2:**
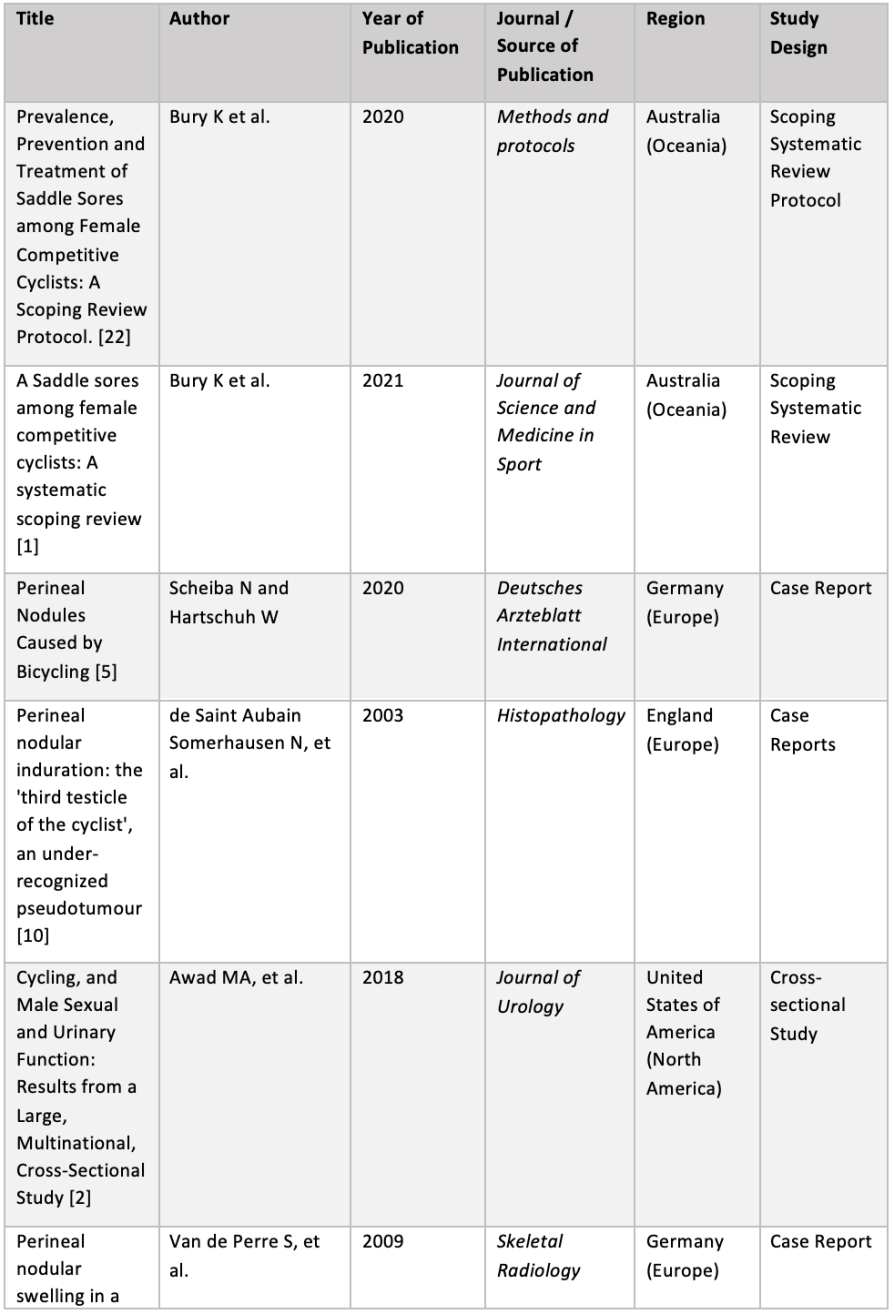

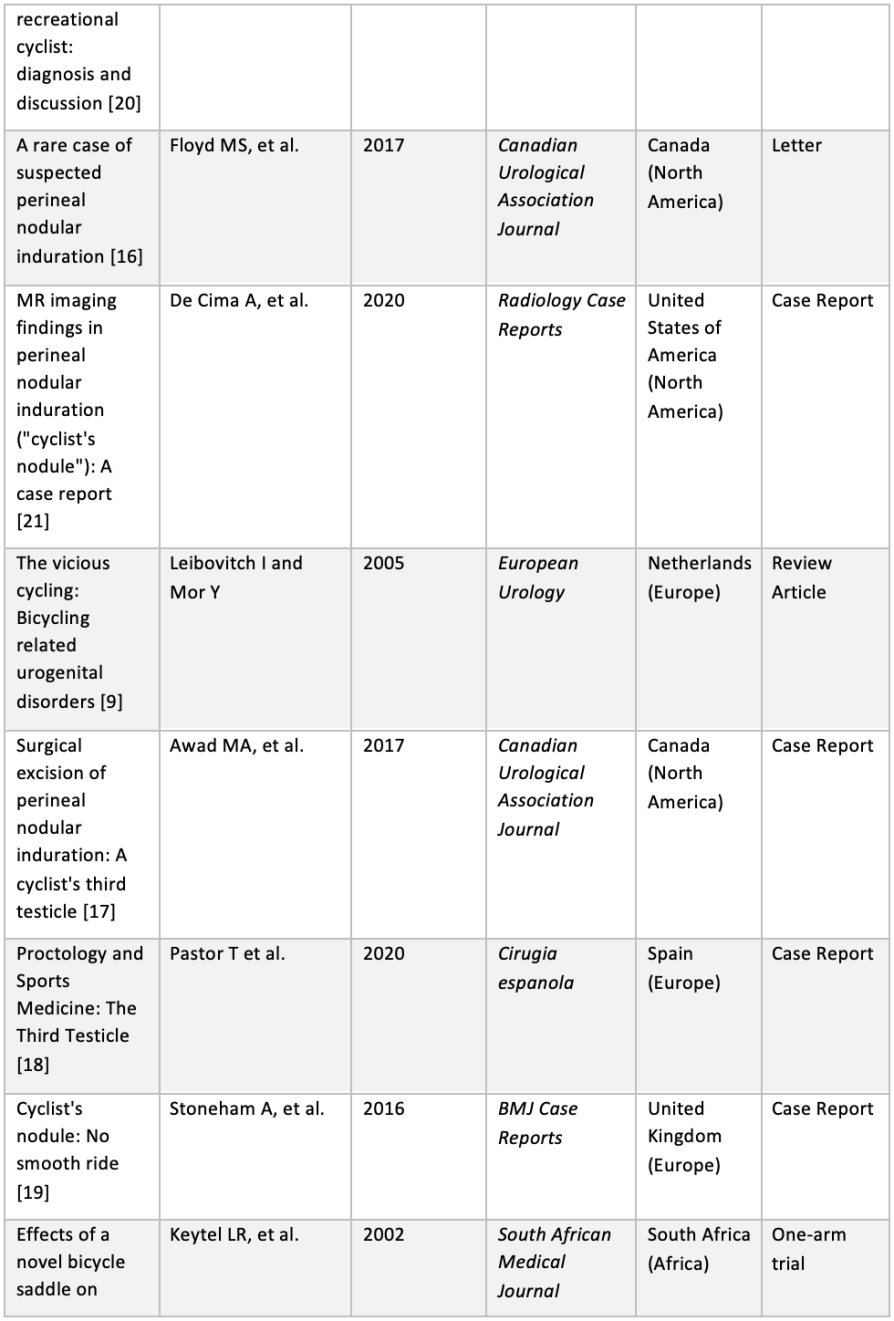

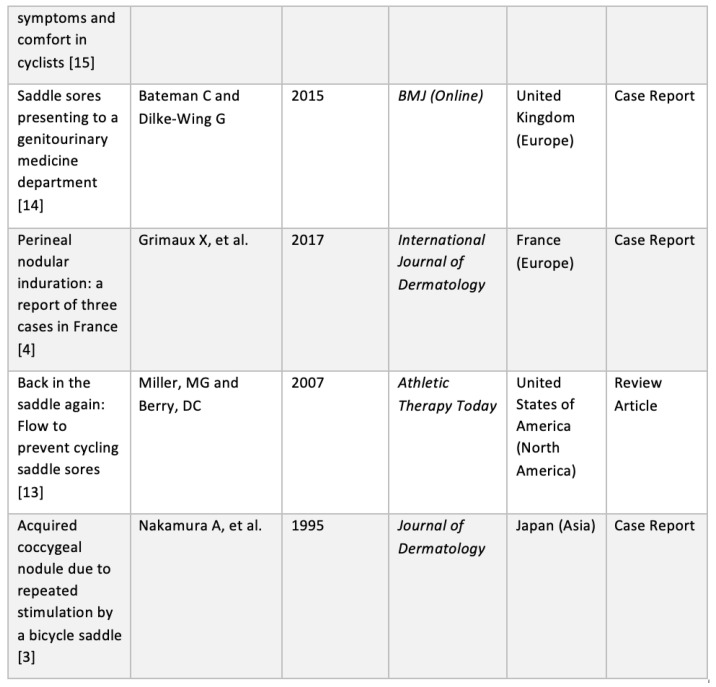
Table of included studies [1,2,3,4,5,9,10,13,14,15,16,17,18,19,20,21,22].

The results were collated, summarised and reported to provide a concise summary of existing research findings on the definition, prevalence, prevention and management of saddle sores, and to identify research gaps. Following protocol, the results were presented as follows:

1. Numerical analysis was used to summarise the data statistically. The demographic characteristics of the included studies were summarised, and outcome measures were grouped and used to assess definition, prevalence, risk factors, prevention and management. The numerical analysis is presented in tabular and graphical form (see Figure 1 and Figure 2, Table 1 and Table 2);
2. Narrative synthesis was used to summarise the main findings of the review. The findings of the studies were grouped into the outcome measures definition, prevalence, risk factors, prevention and management. No assessment of the methodological limitations, risk of bias or meta-analysis of the data took place in this scoping review.

## 3. Results

### 3.1. Overview of Data

The search strategy identified 6978 records across the three databases searched (Figure 1). After removing duplicates, these records were then screened for eligibility using the titles and abstracts, by which 37 were selected for full text review. From these 37, a total of 17 met the inclusion criteria and were included. For the list of studies selected, see Appendix A. The reasons for excluding the 20 articles at the full text review were based on either the concepts in the text not meeting the inclusion criteria, the context of the text meeting the exclusion criteria or no full text being available. The number of studies excluded for each of these reasons is charted in Figure 1—ten studies were excluded because there was no full text available, six because their concept did not focus on saddle sores or PNIs, and four studies because their focus was on urological issues rather than dermatological issues.

### 3.2. Definition

Saddle sores are referred to as lesions affecting the skin in the saddle area, which can be acute or chronic [1,3,4,5,9,10,13,14,15,16,17,18,19,20,21,22]. Acute phase saddle sores present with chafing of the skin and erythema and are associated with infections such as folliculitis and furunculitis [1,9,13]. In the chronic stage, saddle sores are observed to present as nodules with normal overlying skin and fibro-elastic consistency [9,20]. This chronic stage is most commonly referred to as PNI. The variety of names used interchangeably with PNI in the literature reflect how nodules can form in different regions within the saddle area and include ischial hygroma [18], coccygeal nodule [3], cyclist’s nodule [19], accessory testicle [20], third testicle [13] and biker’s nodule [17]. PNIs have been observed to be preceded by saddle sores and increase in size over time in case studies [5,17].

### 3.3. Prevalence

**Figure 3:**
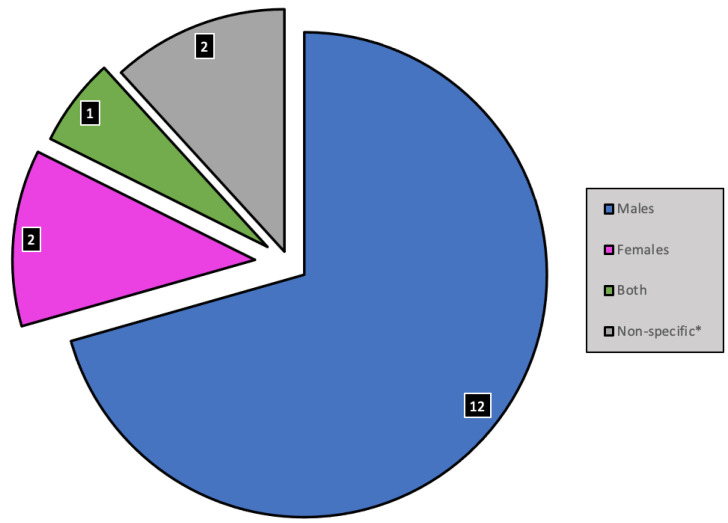
Sex distribution of study population/focus. * Studies that did not refer to sex explicitly within the study population/focus.

Twelve of the studies focused exclusively on saddle sores in males, whereas two focused exclusively on females (see Figure 3). Only one study focused on both genders, while two articles did not explicitly say which sex was in their studies. The patients in case studies were exclusively male, and there was a wide distribution of ages affected by the condition (see Table 1). The youngest case was a 14-year-old boy [3] and the oldest case was a 68-year-old man [10]. There was one cross-sectional study on saddle sore prevalence in male cyclists [2] Male cyclists had significantly higher odds of saddle sores compared to non-cyclists (runners and swimmers) of the same sex [2].

### 3.4. Risk Factors

The most common risk factor cited was cycle duration. Many authors noted that long-distance cyclists were more at risk and this is backed up by case studies [1,4,13,14]. Higher intensity training was also a commonly cited risk factor [9]. Other risk factors included low quality cycling clothing and equipment. Poor saddle and bike design were cited by several studies as putting riders at risk of saddle sores [1,13], although the cross-sectional study included in this review reported that bicycle and saddle type did not have a statistically significant correlation with saddle sores [2], with the same result for saddle angle and street surface.

Poor cycling posture is another reported risk factor. Data from the cross-sectional study showed significantly higher odds of saddle sores when the handlebars were positioned lower than seat height [2] While this study showed no significant correlation between saddle angle and saddle-sore occurrence, one study suggested that pointing the nose of the saddle upwards placed more stress on the perineum, potentially leading to increased risk of saddle sores [13]. Not washing cycling shorts after each use was also cited as a risk factor [14].

### 3.5. Prevention

Most commonly cited prevention was using chamois cream as a lubricant to reduce chafing from seat friction [1,13,14]. Good hygiene and grooming habits were listed as preventative measures in the included studies, including washing cycling shorts after each use [13]. Using a saddle suited to the anatomy of the cyclist was found to reduce saddle symptoms in a one-arm trial [19]. Quality cycling shorts, with a padded area known as a chamois, were recommended by several authors [1,13,14] A previous systematic review documented that removing pubic hair was common advice given in online sources [1].

### 3.6. Treatment/Management

The treatment/management strategies cited in the literature were largely recommended based on the severity of the saddle sore and its associated symptoms. Conservative management was described for small saddle sores that were in the acute phase, up to one week after first appearing. Strategies in this category included hot and cold compresses on the area affected, changing saddle type or wearing cycling shorts with sufficient padding [10,16]. By far the most common conservative strategy was reducing cycling load, including reducing time spent on the bike, distance travelled and training intensity until saddle sores start to resolve [1,5,13,15,16].

Medical interventions for this acute phase included medicated creams to be applied to the affected area. These medicated creams include steroid formulations as well as antifungals and antibiotics. Combinations of steroid and antimicrobial agents (antifungals or antibiotics) were also cited [1]. Antibiotic creams were recommended for use if infection was present [1].

Small saddle sores that needed more urgent treatment or were producing more severe symptoms could be treated with steroid or hyaluronidase injections into the offending lesion(s), although caution needs to be exercised with potential side effects of these treatments [17]. Indeed, one study suggested that skin atrophy attributed to intralesional steroid injections may place patients at risk of recurrence and is something to discuss with patients to gain appropriate informed consent prior to treatment [17].

**Figure 4:**
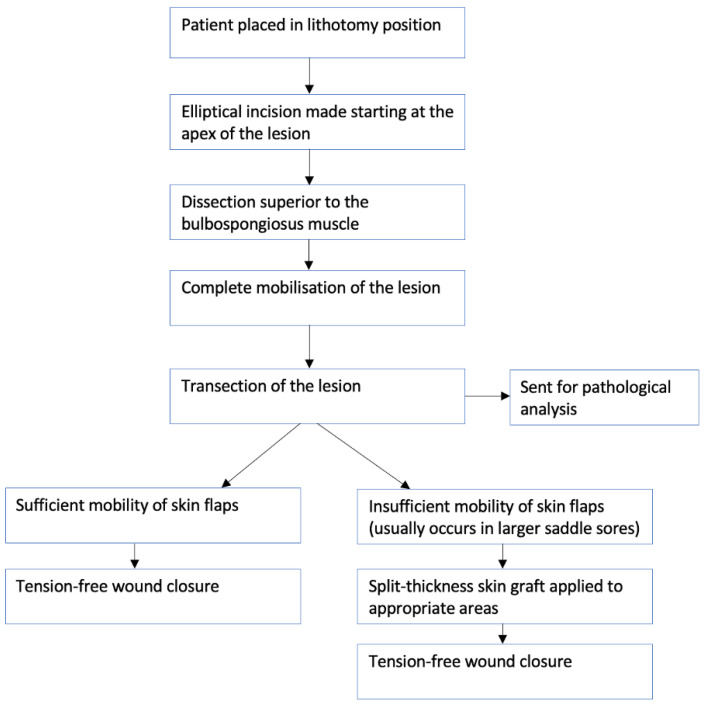
Flow diagram summarising the surgical excision technique reported by Awad et al. [17] in a 48-year-old male cyclist, describing the stages involved.

For larger saddle sores or PNIs, besides conservative management, surgical drainage and/or excision was a recommended option. Only two studies provided follow up analysis post-surgery. One follow-up period was 2 months [17], while the other was unspecified [3]. Neither reported recurrence of saddle-sore lesions post-surgery. The surgical technique for this surgery is described in a case report published by Awad et al. (see Figure 4) [17]. One study reported that surgery should be used cautiously, as scar tissue formation can lead to high recurrence risk [5].

## 4. Discussion

### 4.1. Summary of Results

Only 17 studies met the inclusion criteria, indicating that saddle sores are an underrepresented issue within scientific literature. Females are underrepresented within saddle sore literature [1]. Saddle sores are lesions that affect the skin in the saddle area and have acute and chronic presentations. Chronic saddle sores show variation in nomenclature, with PNI being the most common name used [18]. The most commonly cited risk factor is long-distance cycling [1]. Preventative measures include lubricating creams, hygiene and ergonomic equipment [1,13]. Conservative management largely involves reduction of cycling activity as well as padded shorts. Treatment varies from medicated creams and intralesional steroids for small saddle sores to surgical excision for larger saddle sores. Surgery and steroid use can lead to risk of recurrence.

### 4.2. Definition

With the varied definition of the term saddle sore across the literature, this study sought to summarise the findings and expert opinions from these records to produce a common definition. From the studies identified, it is clear that saddle sores are a dermatological condition with acute and chronic presentations [1,9,10,13]. Precession of PNIs by saddle sores highlights a link between the acute and chronic stage [9,17], where PNIs were reported to increase in size over time and patients continued to cycle frequently [5]. It is proposed that while saddle sores are predisposed to folliculitis and furunculitis, the driving force is primarily the forces of pressure and friction acting between the perineal fascia and pelvic bone during the cycling motion to create repeated skin/dermatology microtrauma, and not infection [9,14].

Saddle sores have been described using a myriad of different terms in the literature [4,5,13,14,15,16,17,18,19,20,21,22]. The term saddle sore is almost universally recognised to describe the acute presentation, and this is backed up by the included literature, as well as the authors’ own experiences of working in this field. With chronic saddle sores, there is much greater variation in the nomenclature used by authors within the literature [5,16,17,18,19] The authors note that in both the medical and cycling community, PNI and associated terms are rarely used and so are poorly recognised. This review highlights a greater need for consistency in the terminology used. Homogenising terminology, with acute and chronic presentations both recognised as saddle sores, is proposed to make understanding of the issue more favourable for people involved in cycling, such as coaching staff and athletes. Ensuring saddle sores are more easily understood and communicated about is aimed to improve dialogue between athletes and medical professionals providing advice and treatment on the topic of saddle sores, helping to address the stigma of discussing saddle issues in cyclists [1,22].

### 4.3. Prevalence

In the literature identified, saddle sores and PNIs were almost exclusively reported in males. The main theme emerging from case studies was that men between 40 and 70 were the most represented (see Table 1). However, we accept that this likely represents publication bias [23] and the author has observed how cyclists of all ages and sexes are affected by saddle sores from experience working in cycling. Younger people can be affected, with one case study describing incidence of the condition in four teenage males in Japan [3].The case studies do not provide any reports of cases in females. Saddle sores are reportedly common in females according to a recent systematic review [1], but this was not evident from the literature, likely representing publication bias [23]. This scoping review has further highlighted the lack of research in female cyclists’ saddle-sore issues and this requires further investigation [1].

In addition, more evidence is needed on whether elite or recreational cyclists are more affected. Professional cyclists were not represented in the case studies, with the majority of cases describing amateur cyclists with a varying degree of participation and intensity of training. The study from Japan mentioned above showed a correlation between commonly cycling to and from school and saddle sores in young males [3]. This may indicate that not wearing appropriate cycling clothing (cycling shorts or leggings with an appropriate chamois, with or without use of a barrier cream) [13], may predispose one to developing saddle sores, but this requires further investigation. Indeed, further research is required into saddle sore prevention.

### 4.4. Risk Factors

The most common risk factor for saddle sores identified in the studies was long distance cycling [1,3,4,13,14]. Two of the patients in a case study included described the initial onset of saddle sores after a particularly long cycle, one of which was reported to be between 200 and 250 km [4]. Expert opinion from a previous systematic review hypothesised that women were at greater risk of saddle sores due to broader hips and an altered pressure distribution in general across the perineal area [1]. Higher intensity cycling was quoted as a risk factor for saddle sores [1], but other risk factors commonly cited in the included in the literature were poor saddle design, cycling posture and saddle hygiene [1,2,14]. However, in a cross-sectional study included in the review, bicycle and saddle type did not show a statistically significant correlation with saddle sores risk [2]. However, handlebar level below seat level in this study was correlated with increased risk of saddle sores [2]. This would suggest that commuter bikes may pose less risk to cyclists than road bikes, as the handlebars are generally higher in relation to the seat. An independent observational study would need to determine which types of saddle place cyclists at greatest risk of saddle sores, for example, racing saddles vs. commuter saddles, as this is not specified. For all the risk factors mentioned, there is a lack of evidence from appropriate observational studies to gauge the magnitude of risk conveyed by each of the factors mentioned.

### 4.5. Prevention

There was a wide variety of preventative measures described in the included literature. These mainly focused on mitigating against risk factors (see Section 3.4). A reduction of activity levels on the bike once a saddle sore develops is inconvenient and frustrating for cyclists, especially at the elite level, causing competition or training time to be missed. Preventative measures should focus on other areas to reduce the overall risk. Using a saddle with superior design and quality was frequently reported to be a preventative measure against saddle sores [1,13,15]. Features of such saddles included adequate cushioning and being adapted to the anatomy of the cyclist [1,15]. In a one-arm trial included in the review, use of a novel saddle more strongly adapted to the anatomy of the rider was shown to reduce saddle symptoms, including saddle sores when compared to a conventional saddle [15]. Further studies need to clarify what design features make a saddle ergonomic. In particular, features that allow a saddle to be suited to an individual’s perineal and pelvic anatomy need to be investigated. A neutral saddle angle was recommended [13], although saddle angle was not statistically correlated with saddle sore risk [2].

Cycling technique or posture is discussed in several papers as a risk factor for saddle sores [2,9,14]. However, different types of cycling lend themselves more to different cycling postures to achieve the greatest performance. An included observational study found that adjusting the handlebar higher than or to the same level of the saddle significantly reduced the odds of saddle sores [2]. More studies are needed to identify other riding habits that can be helpful in reducing the risk further, as well as undertaking studies in both amateur and elite cyclists, which clearly put different demands on cycling technique/posture.

A key causative mechanism targeted by prevention, which is cited in the literature, is friction. The preventative measure used in this case is lubricating cream, more commonly known as chamois cream, to minimise chafing of the skin [1,13] There are several brands of chamois cream mentioned in this included literature [13] It would be important for an independent study to verify their effectiveness. Creams can also include medication such as antibiotics or steroids, but this would largely fall under the category of management, once saddle sores have already affected a cyclist. In the author’s experience, a potential drawback of using these creams is skin pore blockage, which can predispose one to infections described in the literature, such as folliculitis. This needs further investigation.

Appropriate cycling wear is another key pillar of prevention. It is recommended not to wear underwear beneath cycling shorts [13]. Specialised cycling shorts are recommended, but further study is needed to identify exactly which design features of cycling shorts can reduce saddle sores [1,13]. Within the literature, shorts with a chamois (padded crotch section) are recommended, and the shorts should also be loose and breathable to mitigate against perspiration [1,13].

Excellent perineal hygiene is a recommended preventative measure by experts in the field and within the literature [1,13]. The aim of this is to prevent infections associated with saddle sores such as folliculitis and ulcerations. This includes washing cycling shorts after each use, as well as appropriate grooming in the perineal area [13]. There is no evidence provided in studies as to what constitutes ‘proper grooming’. There is an argument that appears in online cycling forums about shaving the area versus not shaving, for the best defence against saddle sores and related folliculitis. This requires further research.

### 4.6. Treatment/Management

Treatment can be largely separated into three categories, conservative, medical and surgical.

Conservative treatment described mostly revolves around a reduction in cycling duration or intensity, and if the saddle sore is severe enough, a break from cycling [1,5,13,15,16]. In the authors experience, this is frustrating for both amateur and elite cyclists, who may wish to train for prolonged periods. Competition schedules and ambition, especially at the elite level, may lead management to escalate quickly from conservative to medical or surgical.

Medicated creams can be used as a preventative measure and also to reduce saddle sore symptoms once they develop. As reported in the literature, medicated creams can take the form of over-the-counter antibacterial, anti-fungal or steroid formulations [1]. Combination formulations are also possible. As such, lubrication is likely to be the factor responsible for the reduction in symptoms achieved using cream and not the presence of the steroid, although the strength of the steroid is mild and stronger steroids may be more effective. More research is needed to provide evidence for the efficacy of antimicrobial creams as well as combinations of these that use steroids as an adjunct. Corticosteroids may be injected into small saddle-sore lesions where the lesion is severe enough to warrant treatment. Caution is recommended with se of corticosteroid and hyaluronidase injections, as the local side effects of steroids can lead to atrophy of the surrounding skin [17]. Skin atrophy may predispose athletes to saddle sore recurrence post-treatment.

For chronic saddle sores, especially those above a certain size and not responding to conservative measures, surgery is the most commonly described treatment option in the literature, especially where the lesions affect a patient’s daily activities and participation in sport. Chronic saddle sores or PNIs often grow larger over time [10,17]. Surgical excision is described as an effective technique amongst authors in appropriate included studies, with no signs of recurrence in the case studies that included follow up [3,17]. A case study that advocated for use of surgery as the gold standard for these types of chronic saddle sores was conducted by surgeons, leaving potential for bias [17]. The evidence on recurrence rates for saddles sores following surgery is conflicting in the included studies. Altered pressure distribution in the perineum following scarring caused by excision surgery is cited to be a reason for high recurrence rates following surgical intervention [5]. One study recommended that surgery be used “sparingly” due to this risk [5]. No adverse sexual or urinary effects were reported in surgical case studies [17]. The maximum follow-up period documented in any surgical case study is 2 months [17] and future studies are necessary with a longer follow-up.

### 4.7. Proposals for Future Saddle-Sore Studies

Further studies have been proposed to address research gaps identified in this review (see Table 2). Studies to assess prevalence and effects of preventions/treatments should be specific to saddle sores to ensure relevance.

### 4.8. Limitations

Limitations include the limited search and lack of assessment of the quality of selected studies or risk of publication bias. The selection of English language papers only, along with exclusion of full text articles not freely available, limited access to all published studies on saddle sores. The inclusion of only one interventional study [15] highlights the need for further randomised controlled trials with the aim of improved prevention and treatment. The search provided limited data on saddle sores in females.

## 5. Conclusions

Saddle sores are an underrepresented issue within scientific literature, particularly in female cyclists. There is a lack of robust evidence available on prevalence, prevention and treatment of saddle sores. This emphasises the need for more studies on saddle sores in the future to provide clinicians with evidence-based information. Further cross-sectional studies are needed to determine true prevalence of saddle sores by age, sex and cycling discipline. Future randomised control trials are recommended to improve knowledge of optimum prevention and treatment. The most common prevention is chamois cream and well fitted cycling equipment, including cycling shorts with a chamois, saddle and bike. Good personal hygiene also plays a role, including grooming habits in the perineal area. Management commonly initially involves rest, which is frustrating to both amateur and elite cyclists. Escalation in size or symptoms can lead to the use of steroid in the form of creams or injections, although care needs to be taken with steroid use, as it can lead to skin atrophy and worsening of pressure sores with time. Larger lesions can be treated by surgical excision. The risk of recurrence reported with surgical and steroid treatment is important to evaluate in future studies to determine if risk outweighs the benefit for patients. The nomenclature used for saddle sores varies for acute and chronic presentations. We recommend consistent use of the terms acute and chronic saddle sores, replacing other terms such as PNI to improve ease of dialogue and understanding regarding the issue amongst clinicians, cyclists and coaches.

## Figures and Tables

**Table 1:** Age profile of patients included in case studies. Note: all case study patients are male.

Age	No. of Patients
<10	0
11–20	4
21–30	0
31–40	0
41–50	4
51–60	4
61–70	3
>70	0

**Table 2:** Proposals for future saddle-sore studies.

Outcome Measure	Proposed Study
Prevalence	Cohort study to assess prevalence of saddle sores in male and female cyclists; Cohort study to assess saddle sore prevalence in different cohorts (e.g., commuters, recreational cyclists, elite level cyclists).
Prevention	Randomised controlled trial to assess effect of chamois cream, simple moisturiser and barrier cream versus no cream (control).
Treatment	Randomised controlled trial assessing effectiveness of moderate and potent steroid cream vs non-medicated lubricant cream; Randomised controlled trial assessing effectiveness of surgery versus conservative management for chronic saddle sores, with long-term follow-up.

**Figure A1:**
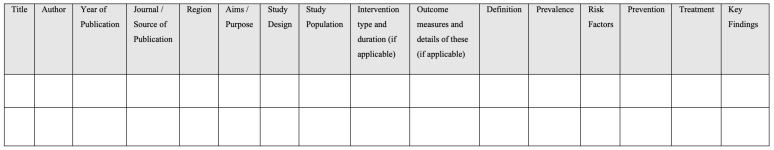
Data charting table template.

## Data Availability

Data is contained within the article.
